# Adhesion and Growth of Vascular Smooth Muscle Cells on Nanostructured and Biofunctionalized Polyethylene

**DOI:** 10.3390/ma6051632

**Published:** 2013-04-29

**Authors:** Katarina Novotna, Marketa Bacakova, Nikola Slepickova Kasalkova, Petr Slepicka, Vera Lisa, Vaclav Svorcik, Lucie Bacakova

**Affiliations:** 1 Department of Biomaterials and Tissue Engineering, Institute of Physiology, Academy of Sciences of the Czech Republic, Videnska 1083, CZ-14220 Prague 4, Czech Republic; E-Mails: k.novotna@biomed.cas.cz (K.N.); marketa.bacakova@biomed.cas.cz (M.B.); lisa.v@biomed.cas.cz (V.L.); 2 Department of Solid State Engineering, Institute of Chemical Technology, Technicka 5, CZ-16628 Prague 6, Czech Republic; E-Mails: nikola.kasalkova@vscht.cz (N.S.K.); petr.slepicka@vscht.cz (P.S.); vaclav.svorcik@vscht.cz (V.S.)

**Keywords:** plasma treatment, biocompatibility, bioactivity, wettability, nanoscale surface roughness, albumin, fibronectin, cell spreading area, tissue engineering

## Abstract

Cell colonization of synthetic polymers can be regulated by physical and chemical modifications of the polymer surface. High-density and low-density polyethylene (HDPE and LDPE) were therefore activated with Ar^+^ plasma and grafted with fibronectin (Fn) or bovine serum albumin (BSA). The water drop contact angle usually decreased on the plasma-treated samples, due to the formation of oxidized groups, and this decrease was inversely related to the plasma exposure time (50–300 s). The presence of nitrogen and sulfur on the polymer surface, revealed by X-ray photoelectron spectroscopy (XPS), and also by immunofluorescence staining, showed that Fn and BSA were bound to this surface, particularly to HDPE. Plasma modification and grafting with Fn and BSA increased the nanoscale surface roughness of the polymer. This was mainly manifested on HDPE. Plasma treatment and grafting with Fn or BSA improved the adhesion and growth of vascular smooth muscle cells in a serum-supplemented medium. The final cell population densities on day 6 after seeding were on an average higher on LDPE than on HDPE. In a serum-free medium, BSA grafted to the polymer surface hampered cell adhesion. Thus, the cell behavior on polyethylene can be modulated by its type, intensity of plasma modification, grafting with biomolecules, and composition of the culture medium.

## 1. Introduction

The use of artificial materials in medicine and biology has become very important, especially their application as substitutes for damaged tissues and organs, or as carriers for drug or gene delivery [1,2,3,4]. These materials have to be biocompatible, *i.e.*, they have to match the mechanical properties of the replaced tissue, and not act as cytotoxic, mutagenic or immunogenic. For specific applications, biocompatible materials can behave as bioinert and not as promoting cell adhesion and growth. For example, materials of these types have been applied in the construction of artificial eye lenses [5] or in the articular surfaces of joint prostheses [6], which are implants requiring transparency or smoothness, and are thus completely cell-free surfaces. Bioinert materials have also been used for fabricating polymeric vascular prostheses in order to prevent the adhesion and activation of thrombocytes and immunocompetent cells on the inner surface of these grafts [7].

However, adhesion, spreading, growth and phenotypic maturation of cells are required for constructing advanced bioartificial tissue replacements, including replacements for blood vessels or for bone. In these replacements, the artificial materials should act as analogs of the natural extracellular matrix, and thus they should support regeneration of the damaged tissue. For example, in vascular tissue engineering, this means that the material should enable reconstruction of the *tunica intima*, formed by a confluent layer of endothelial cells, and also reconstruction of the *tunica media*, which contain vascular smooth muscle cells. For these purposes, advanced artificial materials should not just be passively tolerated by the cells, but should act as bioactive or biomimetic, which means that they induce the required cell responses in a controllable manner (for a review, see [8,9]).

It is generally known that the cell–material interaction is strongly dependent on the physical and chemical properties of the material surface, such as wettability, roughness and topography, or the presence of various chemical functional groups and biomolecules. From this point of view, the surfaces of most materials currently used for constructing tissue replacements are not optimal for integrating with the surrounding tissues, and need further modification in order to improve their physicochemical surface properties and thus enhance the cell colonization and new tissue formation (for a review, see [3,4]). A typical example is provided by synthetic polymers (polyethylene, polypropylene, polystyrene, polyethyleneterephthalate or polytetrafluoroethylene), which are biomaterials widely used in various biotechnologies, including cell cultivation and construction of tissue replacements. In their non-treated state, however, some materials usually form inadequate scaffolds for cell colonization, often due to their relatively high hydrophobicity (water drop contact angle in the range of about 90–120°). These materials can be rendered more hydrophilic by several techniques, particularly by irradiation with ultraviolet (UV) light [10], an ion beam [11,12,13] or exposure to plasma discharge [14,15,16]. In addition to the changes in surface wettability, these treatments also affect the roughness, morphology, electrical conductivity, stability, mechanical properties and chemical composition of the material surface. A common feature of irradiation with UV light, ion beam or plasma treatment is splitting of the bonds within the polymer molecules, which results in the creation of free radicals, double bonds and new functional groups (especially oxygen-containing groups) on the polymer surface. Oxidized groups increase the wettability of polymers, and this supports the adsorption of cell adhesion-mediating extracellular matrix (ECM) molecules in an appropriate spatial conformation, increasing the accessibility of specific sites in these molecules for cell adhesion receptors. Free radicals and unsaturated bonds can then be used for functionalizing the material surface with various biomolecules, e.g., grafting amino acids, oligopeptides or protein molecules, which can also influence (mediate or attenuate) the cell adhesion and growth (for a review, see [3,4]). This grafting occurs spontaneously after exposure of the irradiated material to biological environments, including biological fluids such as blood, intercellular liquid or cell culture media.

Polyethylene has been used in several tissue engineering applications. For example, composites of HDPE with hydroxyapatite [17] or tricalcium phosphate [18] have been developed for the construction of bone replacements, composites of HDPE with graphite for the construction of joint replacements [19], and copolymers of HDPE with hyaluronan for the reconstruction of osteochondral defects [20]. HDPE was also clinically applied for calvarial reconstruction [21]. LDPE is used for producing intravascular catheters for arteriography and angioplasty, due to its advantageous mechanical properties, such as elasticity and flexibility [22].

In our study, two types of polyethylene, namely high density polyethylene (HDPE) and low density polyethylene (LDPE), were modified by Ar^+^ plasma discharge of various exposure times (50, 100 and 300 s), and were subsequently exposed to solutions of two important components of fetal serum, namely fibronectin (Fn) and bovine serum albumin (BSA). We expected the biomolecules from these fluids to graft spontaneously to the plasma-activated polyethylene and to influence cell adhesion and growth, which were studied using rat vascular smooth muscle cells in cultures on these materials. Polyethylene was chosen as a model material for studies of cell–material interaction, due to its easy availability and particularly its relatively simple molecule, consisting only of carbon and hydrogen, which enables clear and reproducible results to be obtained. A comparison was also made for the cell behavior on HDPE and LDPE.

## 2. Results and Discussion

### 2.1. Physical and Chemical Properties of the Polyethylene Samples

#### 2.1.1. Surface Wettability

**Figure 1:**
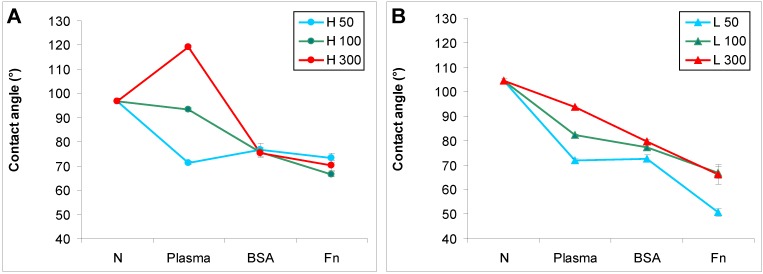
Water drop contact angle on (**A**) HDPE; and (**B**) LDPE in non-treated form (**N**), treated with plasma for 50, 100 or 300 s (**Plasma**), and subsequently grafted with fibronectin (**Fn**) or bovine serum albumin (**BSA**). Mean ± S.D. from 30 measurements performed on 3 samples (10 measurements on each).

The water drop contact angle decreased after plasma modification of the polymers, which means that the surface wettability increased (Figure 1). The highest decrease in the contact angle was observed on samples irradiated for 50 s, and as the exposure time was prolonged the decrease was less apparent. On HDPE irradiated for 300 s, the contact angle even increased (Figure 1A). A similar observation on HDPE was published earlier, and can be explained by the different rearrangement of HDPE after plasma irradiation in comparison with LDPE [23]. It is generally known that the increase in wettability is due to the formation of new oxidized groups on the polymer surface after plasma modification [4,24]. However, surface wettability is also associated with rearrangement of the surface structure, mainly of the oxidized groups, during the aging period of the polymer after plasma exposure. During this period, the oxidized groups are reoriented from the surface to the inside of the polymer. This causes an increase in the contact angle, though in most cases the contact angle of a modified polymer after the aging period still remains lower than the contact angle of the non-treated polymer. The rearrangement of the oxidized groups during aging is influenced by the degree of crosslinking of the polymer structure and the time of plasma modification. A less crosslinked structure and longer modification times lead to greater mobility of these groups. The structure of HDPE is less crosslinked than the structure of LDPE, probably due to the fact that HDPE molecules are more linear and less branched than LDPE molecules, and these differences are further enhanced by plasma irradiation. Thus, the less crosslinked structure of HDPE and relatively long plasma irradiation (300 s) led to higher rotation of the oxidized groups into the polymer, their burrowing into the polymer, their lower exposure on the polymer surface, and thus to lower wettability of the polymer, manifested by a relatively high contact angle (119°), which became even higher than on non-treated HDPE. Moreover, the lesser branching of HDPE creates less opportunity for oxidized groups to occur on this polymer, which might be another important factor influencing the contact angle.

Subsequent grafting of Fn or BSA onto the plasma-activated polymer surface usually resulted in a further decrease in the contact angle. This phenomenon was well apparent, particularly on HDPE and LDPE irradiated with plasma for 100 and 300 s. The increase in surface wettability could be explained by the polar groups present in fibronectin and albumin molecules, oxygen-containing and amine groups. However, on the polymers irradiated for 50 s, the presence of BSA and Fn on an HDPE surface and BSA on an LDPE surface did not have significant effects on the surface wettability, as indicated by the contact angles on these polymers, which were comparable with the values on the samples treated only with plasma. Similar results for BSA on HDPE and LDPE have been published earlier [25,26]. In these studies focused on grafting bioactive substances onto HDPE and LDPE surfaces, only glycin and polyethylene glycol further decreased the contact angle on the plasma-irradiated polymers, while the contact angle remained similar after grafting of BSA, colloidal carbon particles and a combination of BSA and these particles. Similarly, in the present study, grafting the plasma-treated PE with Fn was more efficient in decreasing the contact angle than grafting with BSA, except in the case of HDPE irradiated for 50 s.

#### 2.1.2. XPS Analysis and Immunofluorescence Staining of Grafted Biomolecules

Fibronectin and albumin are proteins which contain nitrogen and disulfide bonds [27,28]. The presence of these biomolecules on the PE surface was therefore tested using X-ray photoelectron spectroscopy analysis (XPS) by determining the concentration of nitrogen and sulfur on the polymer surface. XPS also determined the concentration of oxygen as evidence that oxidized polar groups were formed after plasma irradiation.

XPS analysis showed that the concentration of nitrogen was significantly higher on HDPE and LDPE activated with plasma and subsequently grafted with Fn and BSA than on samples only treated with plasma (Table 1). The nitrogen found in a low amount on the plasma-treated samples was probably bound from the ambient atmosphere after plasma exposure. In addition, sulfur was determined on the samples grafted with Fn and BSA. Due to the higher presence of nitrogen and sulfur on the grafted samples than on the only plasma-treated samples, we can conclude that fibronectin and albumin were successfully bound on the plasma-treated polymer surface. XPS also indicates that the concentration of oxygen increased after the polymer was exposed to plasma, and this led to a decline in the contact angle (Figure 1). The higher concentration of oxygen on the irradiated samples than on the non-treated samples indicated the formation of newly oxidized polar groups after plasma modification.

**Table 1:** Concentration (in at. %) of carbon (C), oxygen (O), nitrogen (N) and sulfur (S), determined by XPS analysis, on HDPE and LDPE treated with plasma for 50 s (**50**), and subsequently grafted with fibronectin (**Fn**) and bovine serum albumin (**BSA**). Mean from three measurements for each experimental group; the measurement error did not exceed 5%.

**Material/Modification**	**HDPE**				**LDPE**			
	C (at. %)	O (at. %)	N (at. %)	S (at. %)	C (at. %)	O (at. %)	N (at. %)	S (at. %)
50	82.2	14.2	2.5	0	83.0	14.8	1.5	0
50 Fn	72.9	20.2	6.7	0.2	82.2	13.6	2.8	at limit of detection
50 BSA	74.4	15.3	9.9	0.4	72.7	15.3	10.9	0.4

**Figure 2:**
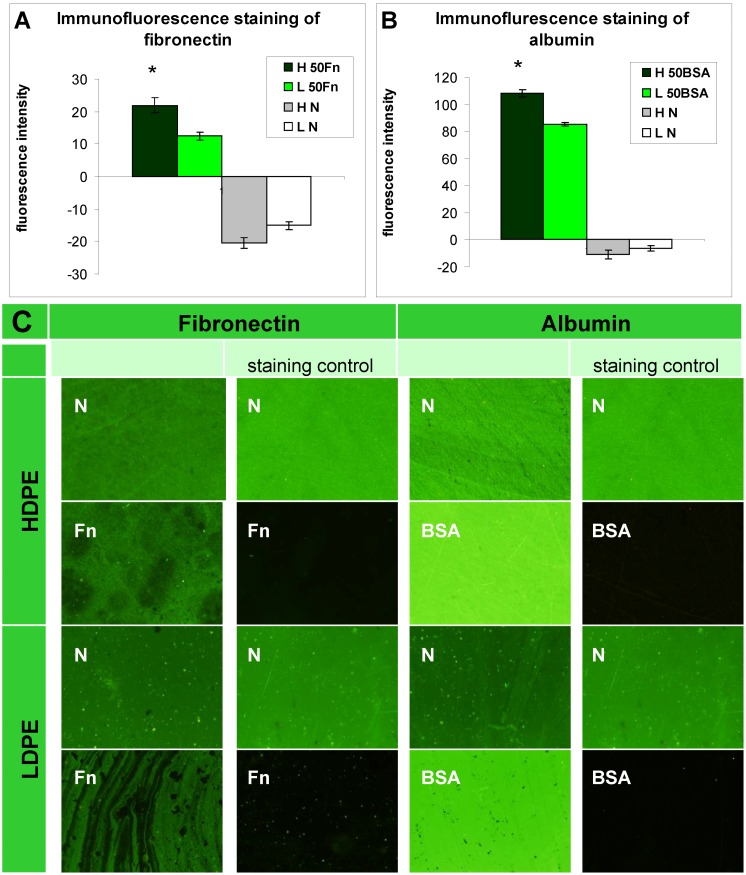
Immunofluorescence staining of (**A**) fibronectin; and (**B**) albumin and their microphotographs (**C**) on HDPE (**H**) and LDPE (**L**), in non-treated form (**N**), treated with plasma for 50 s and subsequently grafted with fibronectin (**50 Fn**) or bovine serum albumin (**50 BSA**). Olympus IX 51 microscope, obj. 10x, DP 70 digital camera. The measurements were performed at the same exposure time for all experimental groups (1.2 s).

The presence of fibronectin and albumin was also tested by immunofluorescence staining (Figure 2). The resultant fluorescence intensity was presented as the difference between the fluorescence intensity measured on samples treated with both primary and secondary antibodies and on samples treated with the secondary antibody only (*i.e.*, the staining control). On HDPE and on LDPE grafted with Fn or BSA, the fluorescence intensity reached high positive values, indicating the presence of Fn and BSA on the polymer surface. By contrast, the fluorescence intensity had negative values on the non-treated polymers [Figure 2(A,B)]. This was probably due to non-specific binding of the secondary antibody to the polymer surface, because the fluorescence intensity of the staining control, *i.e.*, the sample incubated only in the secondary antibody, was higher than the fluorescence intensity after staining the non-treated polymer with both primary and secondary antibodies (Figure 2C). The non-specific binding of the secondary antibody on samples grafted with Fn and BSA was significantly reduced due to the presence of these biomolecules. In this context it should be pointed out that in studies using immunocytochemical methods, BSA [25,26,29], the whole blood serum [30] or solutions of other biomolecules, e.g., gelatin [12] are commonly used for blocking non-specific binding sites for antibodies. In addition, the fluorescence intensity on HDPE grafted with Fn or BSA was higher than on LDPE, which showed that Fn and BSA were grafted in a higher concentration on the HDPE surface than on LDPE [Figure 2(A,B)].

#### 2.1.3. Surface Roughness and Morphology

**Figure 3:**
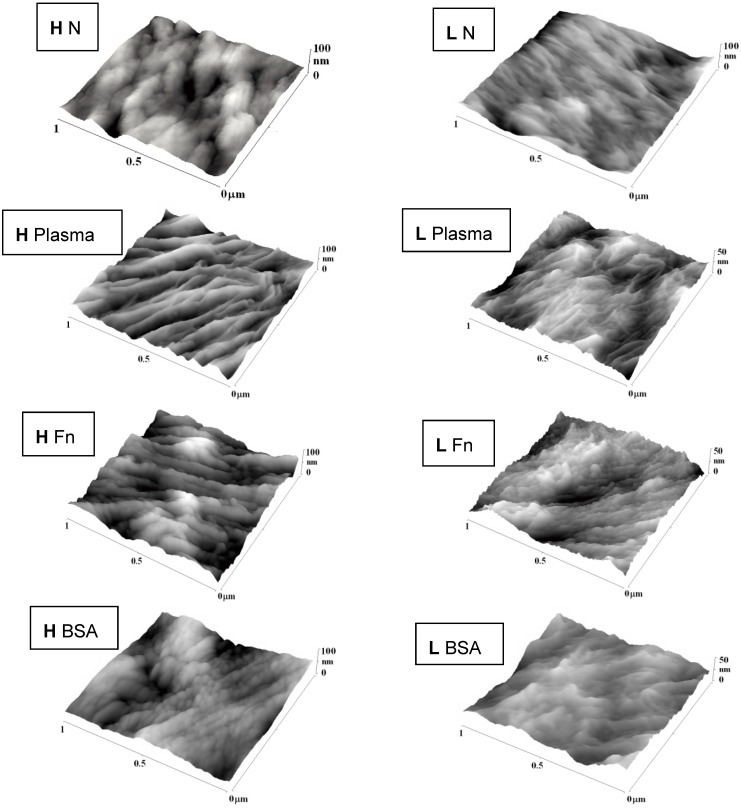
AFM images of HDPE (**H**) and LDPE (**L**) in non-treated form (**N**), treated in plasma for 50 s, and subsequently grafted with fibronectin (**Fn**) or bovine serum albumin (**BSA**).

Changes in surface roughness and morphology after plasma exposure and grafting with biomolecules were studied by the Atomic Force Microscopy (AFM) method (Figure 3, Table 2). Plasma treatment led to ablation of the surface layer [23], which increased the nanoscale surface roughness in proportion to the exposure time. These events were more apparent on HDPE than on LDPE, and could be explained by the different structure of the two polymers. For example, LDPE is more crosslinked than HDPE, and thus it could be more resistant to ablation. In our earlier study, plasma treatment of PE for 400 s resulted in the removal of a surface layer of HDPE that was about 1 μm in thickness, but of a layer of LDPE that was only 0.6 μm in thickness [23].

**Table 2:** The mean surface roughness values (R_a_) of LDPE and HDPE in non-treated form (**N**), treated in plasma for 50, 100 or 300 s (**50, 100, 300**), and subsequently grafted with fibronectin (**Fn**) or albumin (**BSA**).

Sample	HDPE	LDPE
**N**	6.1	5.0
**50**	7.4	4.7
**50** Fn	10.4	5.5
**50** BSA	6.9	5.4
**100**	8.4	6.7
**100** Fn	11.7	4.6
**100** BSA	9.4	4.7
**300**	11.5	6.6
**300** Fn	12.0	5.6
**300** BSA	7.8	4.6

In addition, the surface morphology of both types of PE was changed after plasma modification. A lamellar structure appeared on the polymer surface, perhaps due to the different ablation rate of the amorphous and crystalline phases. The amorphous phase is ablated preferentially [23,31]. The newly created lamellar structure was more apparent on the HDPE surface, while the LDPE surface appeared to be ablated more uniformly (homogeneously). The reason could be a higher proportion of the crystalline phase in HDPE, while the amorphous phase prevails in LDPE [23].

Subsequent grafting with Fn or BSA further changed the surface roughness (see Table 2) and the morphology of PE. Grafting with Fn strongly increased the surface roughness of HDPE, while BSA did not significantly increase, or even reduced, the surface roughness of the plasma-treated surface, although this roughness still remained higher than on the non-treated polymer. The lamellar structure was less apparent on the grafted surface than on the only-treated HDPE surface. Grafting LDPE with Fn or BSA produced rather small and non-significant changes in surface roughness and morphology. On LDPE irradiated for 100 or 300 s, both Fn and BSA showed a tendency to smoothen the material surface (Figure 3).

### 2.2. Cell Adhesion and Growth

#### 2.2.1. Cell Adhesion and Growth on LDPE and HDPE Samples in a Standard Serum-supplemented Medium

**Figure 4:**
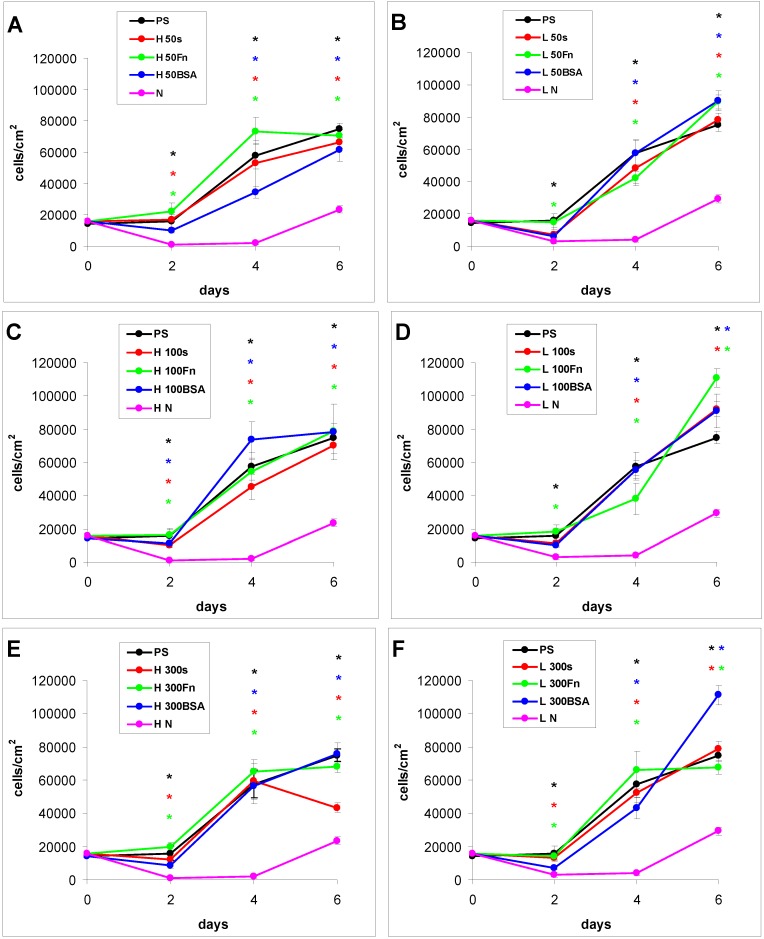
Growth curves of rat aortic smooth muscle cells cultured in a serum-supplemented medium on HDPE (**A–C**) or LDPE (**D–F**) in non-treated form (**N**), treated with plasma for 50, 100 or 300 s (**50, 100, 300**), and subsequently grafted with fibronectin (**Fn**) or bovine serum albumin (**BSA**). A standard cell culture polystyrene dish (**PS**) was used as a reference material. Mean ± S.E.M. from 20 measurements on microphotographs (day 2 and 4) or 200 measurements on a ViCell XR Analyser (Beckman Coulter, Brea, CA, USA) (day 6). ANOVA, Student–Newman–Keuls Method. Statistical significance: * *p* ≤ 0.05 compared to non-modified HDPE or LDPE.

The proliferation of cells in DMEM with fetal bovine serum (FBS) had in general a growing tendency. On day 2 after seeding, there were similar numbers of initially adhered cells on the control polystyrene, on the samples exposed to a plasma discharge, and on all Fn-grafted and BSA-grafted samples (Figure 4). This cell behavior was observed on both LDPE and HDPE, and could be explained by a masking effect of the serum in the culture medium, *i.e.*, secondary adsorption of serum components influencing cell adhesion, particularly vitronectin, fibronectin and albumin on the samples, which occurs within a few minutes after these substrates are exposed to the culture medium (for a review, see [3,4,9]). Significant differences were found only between the non-treated samples and some modified samples, usually with the exception of the BSA-grafted samples [Figure 4(A,B,D–F)], and in two cases also with the exception of the samples only irradiated with plasma [Figure 4(B,D)]. 

From day 2 to day 4 after seeding, the cells on all modified PE samples showed an intense increase in their growth, while the cell growth on the non-treated PE samples was stagnant. As a result, the cell numbers on all modified samples on day 4 were significantly higher than the values on the non-treated samples. However, the differences among the groups of modified samples still remained non-significant (Figure 4).

From day 4 to day 6 after seeding, the cells on most of the HDPE samples reached confluence, slowed down their proliferation and entered the stationary growth phase, *i.e.*, their number did not increase as rapidly as between day 2 and day 4, and on some samples the number even decreased. By contrast, the cells on the LDPE samples usually continued their growth, and thus reached on an average higher final cell population densities. On day 6 after seeding, these densities ranged from about 78,000 to 111,000 cells·cm^−2^ on LDPE, while there were only 43,000 to 79,000 cells·cm^−2^ on HDPE (Figure 4).

**Figure 5:**
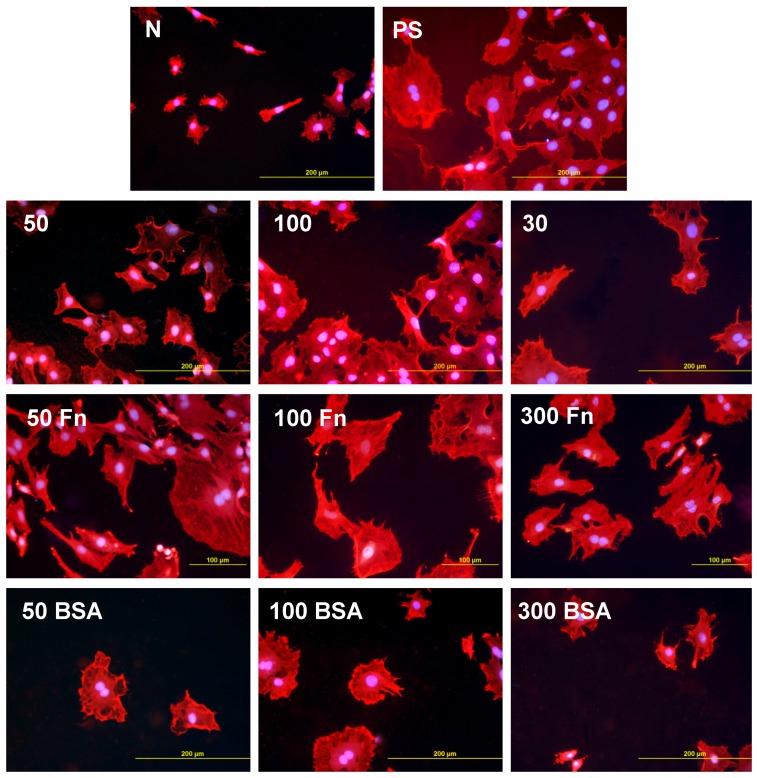
Morphology of rat aortic smooth muscle cells in two-day-old cultures in a serum-supplemented medium on HDPE in non-treated form (**N**), treated with plasma for 50, 100 or 300 s (**50, 100, 300**), and subsequently grafted with fibronectin (**Fn**) or bovine serum albumin (**BSA**). A standard cell culture polystyrene dish (**PS**) was used as a reference material. Cells stained with Texas Red C_2_-Maleimide and Hoechst #33342. Olympus IX 51 microscope, obj. 20, DP 70 digital camera, bar 200 μm or 100 μm (Fn-grafted samples).

**Figure 6:**
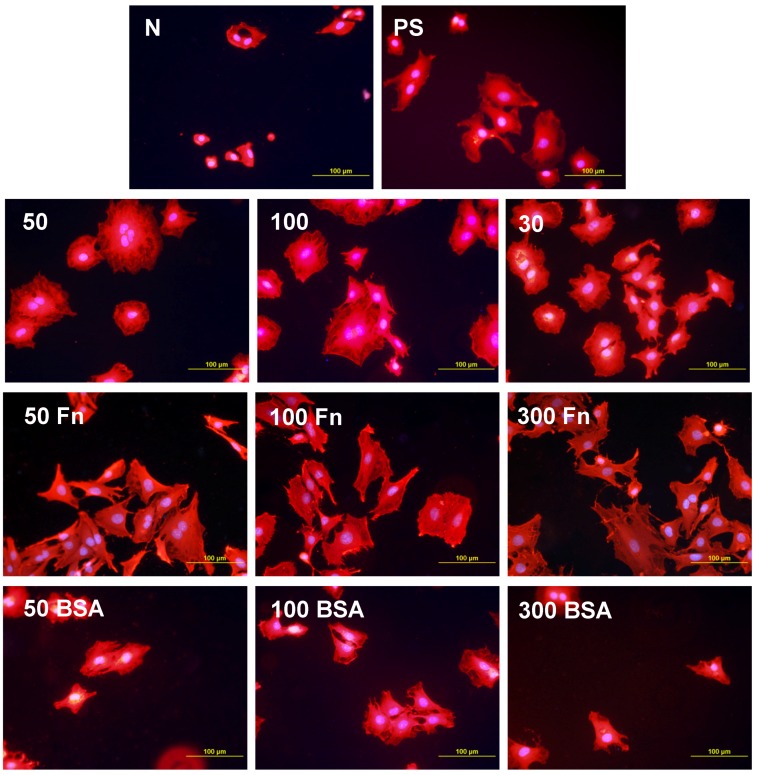
Morphology of rat aortic smooth muscle cells in two-day-old cultures in a serum-supplemented medium on LDPE in non-treated form (**N**), treated with plasma for 50, 100 or 300 s (**50, 100, 300**), and subsequently grafted with fibronectin (**Fn**) or bovine serum albumin (**BSA**). A standard cell culture polystyrene dish (**PS**) was used as a reference material. Cells stained with Texas Red C_2_-Maleimide and Hoechst #33342. Olympus IX 51 microscope, obj. 20, DP 70 digital camera, bar 200 μm or 100 μm (Fn-grafted samples).

**Figure 7:**
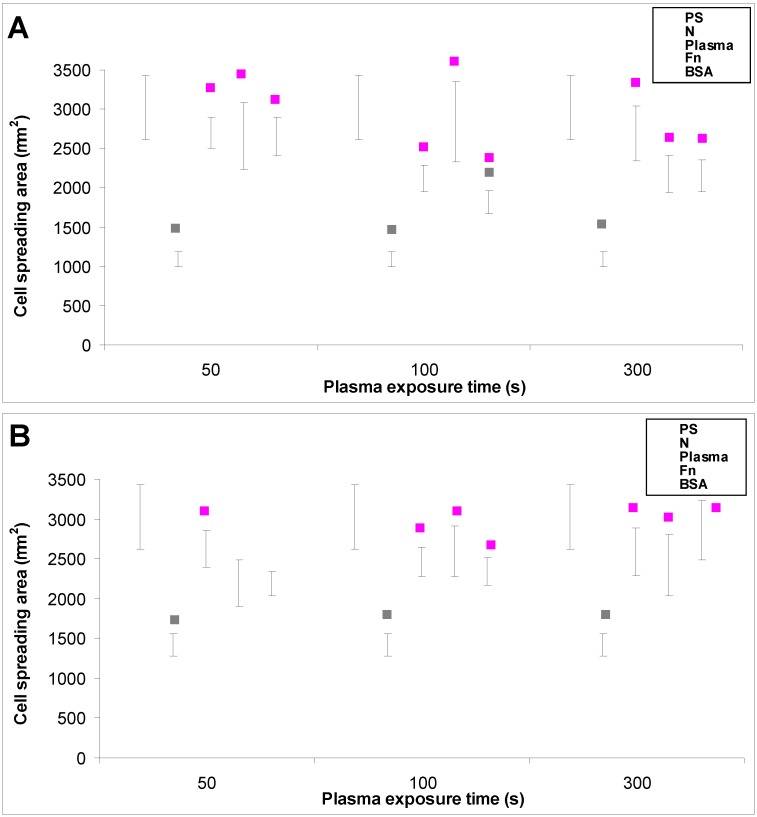
The size of the cell spreading area of rat aortic smooth muscle cells in two-day-old cultures in a serum-supplemented medium on (**A**) HDPE; or (**B**) LDPE in non-treated form (**N**), treated with plasma for 50, 100 or 300 s, and subsequently grafted with fibronectin (**Fn**) or bovine serum albumin (**BSA**). A standard cell culture polystyrene dish (**PS**) was used as a reference material. Mean ± S.E.M. from 32 to 95 measured cells for each experimental group. ANOVA, Student–Newman–Keuls Method. Statistical significance: ▪ *p* ≤ 0.05 compared to other experimental groups, indicated by the colors of these groups above the columns.

Particularly the cells on the BSA-grafted LDPE samples continued their exponential growth, and on day 6 after seeding their population densities were among the highest values reached on these samples, especially on LDPE activated with plasma for 300 s (Figure 4F). These results can be considered as surprising, because albumin is known as a cell non-adhesive molecule [32], which has been used for creating bioinert cell-free surfaces, applicable e.g., in blood-contacting devices [33,34]. In our study, the non-adhesive properties of albumin are slightly reflected by the relatively low numbers of initially adhered cells on the BSA-grafted samples, which often did not differ significantly from the values on the non-treated polyethylene (Figure 4). Nevertheless, the cell spreading areas were mostly larger than in the cells on non-treated samples, and usually not significantly smaller than on the control polystyrene dishes, except BSA-grafted HDPE treated with plasma for 100 s (Figure 5, Figure 6 and Figure 7). Albumin has been reported to promote adsorption of ECM molecules, e.g., vitronectin and fibronectin, in advantageous spatial conformations, supporting the accessibility of these molecules by cell adhesion receptors (for a review, see [25]). These molecules cannot only be adsorbed over the albumin from the serum of the culture medium, but are also synthesized and deposited by VSMC themselves [35], especially at later culture intervals. This may account for the relatively high proliferation activity of the cells on BSA-grafted PE (Figure 4).

As for the non-treated HDPE and LDPE samples, the cell number increased from day 4 to 6 on these materials (Figure 4). However, the maximum population densities on these polymers still remained significantly lower (from about 24,000 to 29,000 cells·cm^−2^) than on the modified samples (from 43,000 to 111,000 cells·cm^−2^).

In accordance with the weak cell proliferation on non-treated PE, the visualization of the cells on day 2 also showed a lower size of the cell spreading area on non-treated PE than on the modified samples (Figure 5, Figure 6 and Figure 7). This insufficient cell spreading can be explained by the relatively high hydrophobicity of non-treated HDPE and LDPE. It is known that, on highly hydrophobic surfaces, the cell adhesion-mediating proteins, e.g., fibronectin and vitronectin, present in the serum of the culture medium, are adsorbed in rigid, non-physiological and denatured form, less appropriate for binding the specific sites in the protein molecules by the cell adhesion receptors (for a review, see [3,4,9]). By contrast, the modified polymers were more hydrophilic, with an enhanced nanostructure or grafted with biomolecules. As mentioned above, BSA supports the adsorption of cell adhesion-mediating molecules in a near-physiological conformation, appropriate for binding to the cell adhesion receptors. In addition, the fibronectin grafted on some samples provided the possibility for direct binding of the cells, e.g., through specific RGD-containing amino acid sequences in the Fn molecules [28].

#### 2.2.2. Cell Adhesion and Growth on LDPE and HDPE in a Serum-Free Medium

**Figure 8:**
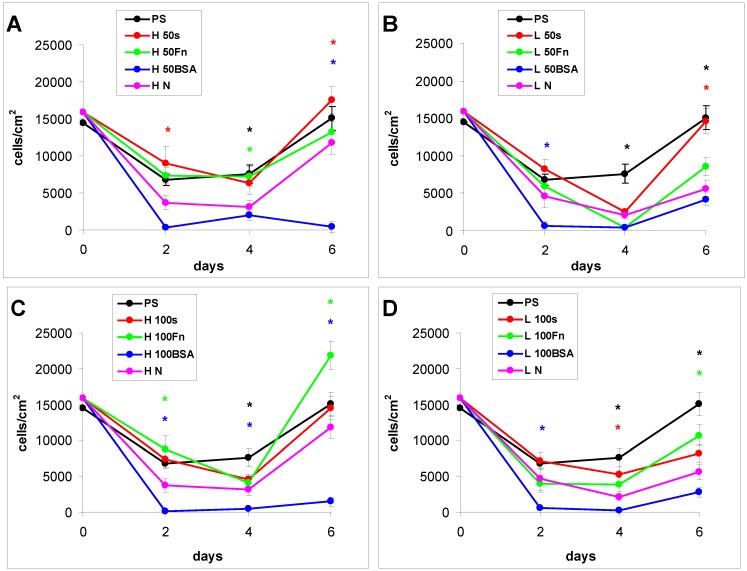

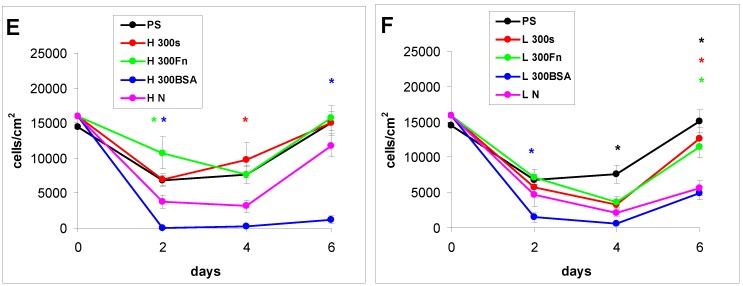
Growth curves of rat aortic smooth muscle cells cultured in a serum-free medium on (**A–C**) HDPE; or (**D–F**) LDPE in non-treated form (**N**), treated with plasma for 50, 100 or 300 s (**50, 100, 300**), and subsequently grafted with fibronectin (**Fn**) and bovine serum albumin (**BSA**). A standard cell culture polystyrene dish (**PS**) was used as a reference material. Mean ± S.E.M. from 20 measurements (day 2 and 4) or 4 measurements (day 6). ANOVA, Student–Newman–Keuls Method. Statistical significance: * *p* ≤ 0.05 compared to the non-modified LDPE or HDPE.

In the serum-free medium, the differences in the behavior of VSMC on the PE samples with various modifications became more apparent than in the serum-supplemented medium, and the growth dynamics of the cells revealed a different trend. From days 2 to 4 after seeding, the cell number was mostly stagnant, and on some samples it even decreased. There was an increasing tendency only between days 4 and 6 (Figure 8). This stagnancy in cell growth was most likely because there was a limited quantity of the ECM proteins normally present in fetal serum, mainly vitronectin and fibronectin, which ensure proper adhesion, spreading and subsequent growth of cells on the material surfaces. The serum-free medium used in this study did not contain these proteins. However, cell adhesion-mediating proteins can be present on cells in small quantities even after trypsinization [36], and could be used for cell adhesion after seeding cells on the tested materials, particularly on polystyrene, non-treated PE and plasma-treated PE (on Fn-grafted PE, the Fn molecules could be used directly for cell adhesion). In addition, VSMC are able to synthesize a wide spectrum of cell adhesion-mediating ECM molecules (for a review, see [35]). This takes place especially at later culture intervals. By way of these molecules, the cells adhere to the material surface through the bonds between specific amino acid sequences in these molecules and cell adhesion receptors on the cell membrane, e.g., integrins. Another way for cells to attach to the tested materials is by so-called weak chemical bonding, *i.e.*, hydrogen bonding, electrostatic, polar or ionic interactions between various molecules on the cell membrane and functional chemical groups on the polymers, *etc*., but this type of binding does not transfer specific signals into the cells and does not ensure their survival, growth and other functions (for a review, see [3,4,9,37,38]).

**Figure 9:**
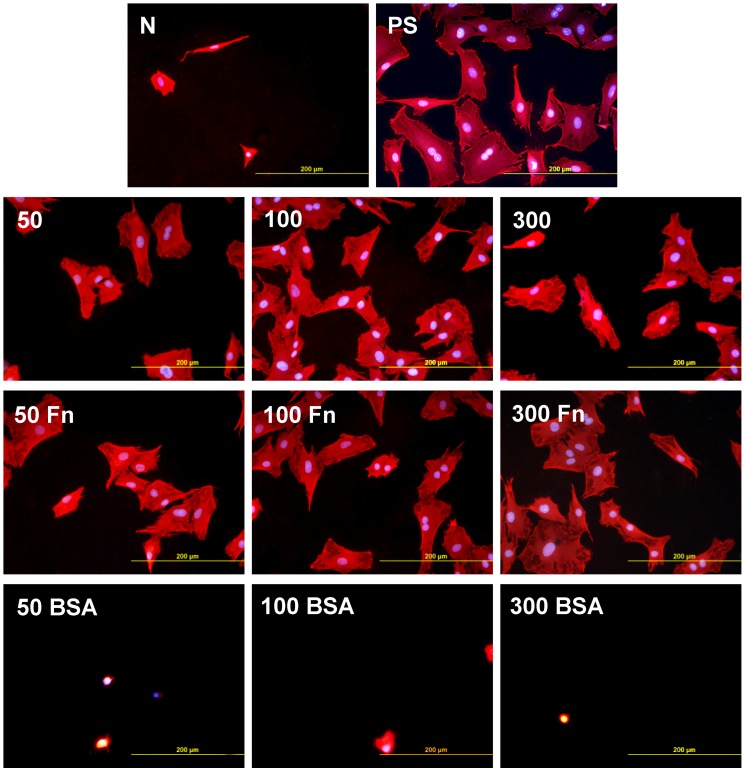
Morphology of rat aortic smooth muscle cells in two-day-old cultures in a serum-free medium on HDPE in non-treated form (**N**), treated with plasma for 50, 100 or 300 s (**50, 100, 300**), and subsequently grafted with fibronectin (**Fn**) and bovine serum albumin (**BSA**). A standard cell culture polystyrene dish (**PS**) served as a reference material. Cells stained with Texas Red C_2_-Maleimide and Hoechst #33342. Olympus IX 51 microscope, obj. 20, DP 70 digital camera, bar 200 μm or 100 μm (Fn-grafted samples).

**Figure 10:**
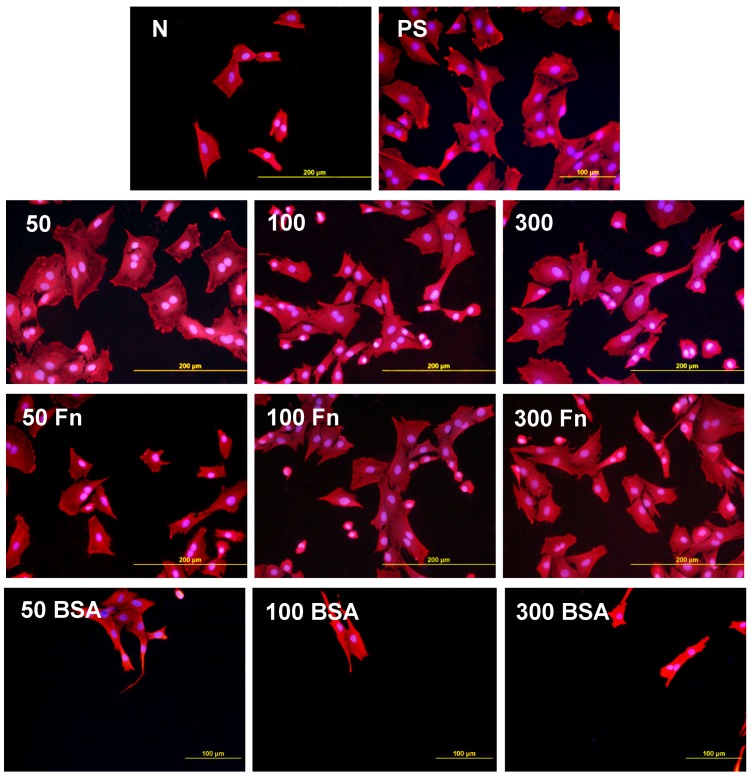
Morphology of rat aortic smooth muscle cells in two-day-old cultures in a serum-free medium on LDPE in non-treated form (**N**), treated with plasma for 50, 100 or 300 s (**50, 100, 300**), and subsequently grafted with fibronectin (**Fn**) and bovine serum albumin (**BSA**). A standard cell culture polystyrene dish (**PS**) was used as a reference material. Cells stained with Texas Red C_2_-Maleimide and Hoechst #33342. Olympus IX 51 microscope, obj. 20, DP 70 digital camera, bar 200 μm or 100 μm (Fn-grafted samples).

As early as on day 2 after seeding, the cell non-adhesive properties of BSA were completely revealed in the serum-free medium. This effect of albumin was more apparent on HDPE, where the cell densities ranged from 37 to 367 cells·cm^−2^, while on LDPE the values were slightly higher (551 to 1470 cells·cm^−2^; Figure 8). Accordingly, the cell spreading was also the lowest on the HDPE samples (Figures 9). The cells mainly assumed a spherical shape on these surfaces, while on the other samples, even on the BSA-grafted LDPE samples (Figure 10) or on non-treated LDPE and HDPE, the cell morphology was spindle-shaped or polygonal.

From day 4 to 6, the cell numbers increased slightly, probably by preferential cell growth on sites with discontinuity of the albumin layer, or secondary deposition of cell adhesion-mediating ECM proteins synthesized by the cells themselves on the albumin layer. On day 6, this increase was slightly more apparent on BSA-grafted LDPE (from 2800 to 4900 cells·cm^−2^) than on BSA-grafted HDPE (from 400 to 1500 cells·cm^−2^). However, these samples still displayed the lowest cell population densities, irrespective of plasma exposure time (Figure 8).

**Figure 11:**
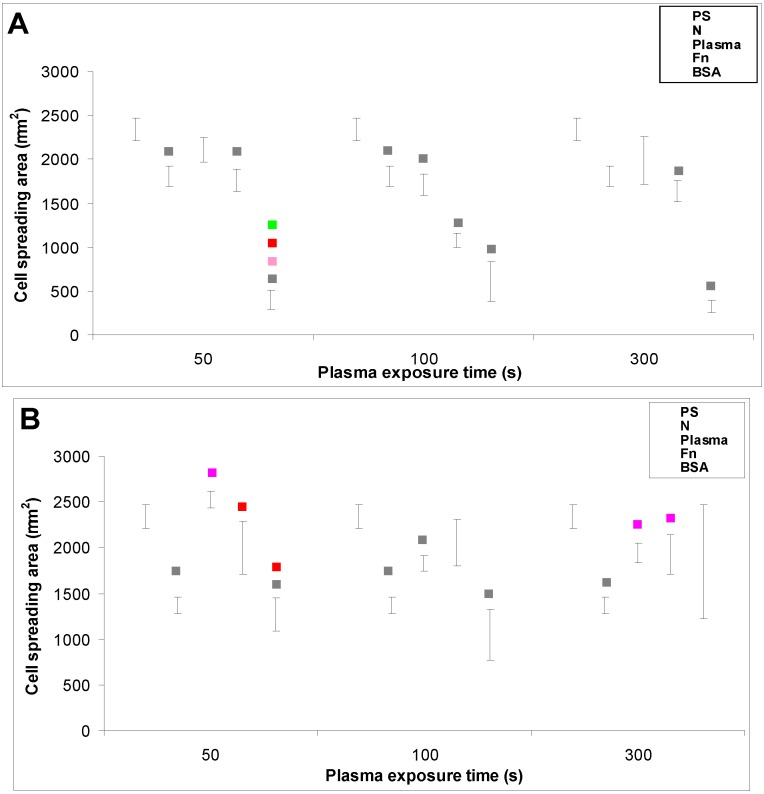
The size of the cell spreading area of rat aortic smooth muscle cells in two-day-old cultures in a serum-free medium on (**A**) HDPE; or (**B**) LDPE in non-treated form (**N**), treated with plasma for 50, 100 or 300 s, and subsequently grafted with fibronectin (**Fn**) or bovine serum albumin (**BSA**). A standard cell culture polystyrene dish (**PS**) was used as a reference material. Mean ± S.E.M. from 4 to 269 measured cells. ANOVA, Student–Newman–Keuls Method. Statistical significance: ▪ *p* ≤ 0.05 compared to other experimental groups, indicated by the colors of these groups above the columns.

The effects of the grafted fibronectin were controversial. We expected high adhesion and subsequent growth of cells on these samples, due to specific receptor-mediated adhesion of cells to Fn grafted on the plasma-activated polymer surfaces. However, the cell densities in general on all Fn-grafted samples were similar to the values on the plasma-irradiated surfaces, or on the control polystyrene culture dishes. Among all Fn-grafted samples, the weakest proliferation was observed on LDPE irradiated with plasma for 50 s (L 50Fn; Figure 8B), where the cell population density on day 4 was lower than on non-treated LDPE, and as low as on the BSA-grafted samples. On day 6, the number of cells increased, but was still significantly lower than on PS or LDPE irradiated for 50 s (Figure 8B). The relatively low cell proliferation activity on Fn-grafted polyethylene could be explained by relatively low quantity of the Fn molecules attached to the polymer surface. As indicated by XPS, the concentrations of nitrogen and sulfur, which are indicators of the presence of protein molecules, were relatively low on the Fn-grafted surfaces in comparison with the BSA-grafted surfaces, particularly on the LDPE samples. On LDPE, the concentration of S was even on the limit of detection (Table 1). In accordance with this, the intensity of fluorescence of antibody-labeled Fn grafted to plasma-activated surfaces is lower than in the case of albumin [Figure 2(A,B)] In addition, the pictures indicate less homogeneous distribution of fibronectin on these surfaces compared to albumin (Figure 2C). Also the geometrical conformation of Fn, which is more important for cell binding than the absolute amount of Fn immobilized on the material surface ([39], for a review, see [3,4,9]) could be less appropriate for binding by the cell adhesion receptors. In spite of this, the cells on the Fn-grafted HDPE and LDPE samples were of similar polygonal morphology as the cells on the control polystyrene and plasma-irradiated samples (Figure 9 and Figure 10), although their spreading area was often smaller (Figure 11).

## 3. Experimental Section

### 3.1. Preparation and Modification of Polymer Samples

The experiments were carried out on high-density and low-density polyethylene foils (HDPE and LDPE, both purchased from Granitol A. S., Moravsky Beroun, Czech Republic). The HDPE foils were of the Microten M*S type (thickness 40 μm, density 0.951 g·cm^−3^, melt flow index 0.14 g·10 min^−1^), and the LDPE was of the Granoten S*H type (thickness 40 μm, specific density 0.922 g·cm^−3^, melt flow index 0.8 g·10 min^−1^). Both types of polyethylene were cut into circular samples (diameter 2 cm) using a metallic perforator (punch).

The samples were then treated by an Ar^+^ plasma discharge (gas purity 99.997%) using a Balzers SCD 050 device (BalTec Maschinenbau AG, Pfäffikon, Switzerland). The time of exposure was 50, 100 or 300 s, and the discharge power was 3 W. Immediately after the plasma treatment, the samples were immersed in solutions of two components of fetal bovine serum (FBS), namely 2 wt.% of fibronectin (Fn, Sigma-Aldrich, St. Louis, MI, USA, Cat. No. F2006) or 2 wt.% of bovine serum albumin (BSA, Sigma-Aldrich, Cat. No. A9418), both diluted in phosphate-buffered saline (PBS; Sigma-Aldrich, Cat. N° P4417). The samples were incubated for 24 h at room temperature (RT) in order to allow grafting of biomolecules to the plasma activated polymer surface. The samples were then rinsed in distilled water, air-dried at RT and stored in an air atmosphere for three weeks in order to reorganize and stabilize their surface structure, particularly the orientation of the oxidized structures on the material surface (the so-called aging period) [23].

### 3.2. Characterization of the Physical and Chemical Properties of the Polymer Surface

#### 3.2.1. Surface Wettability

The sessile water drop contact angle was measured by See System reflection goniometry (Masaryk University, Brno, Czech Republic). The volume of the water drop on the polymer surface was 8 μL. The contact angle was measured after an aging period of three weeks. For each experimental group, three samples were used, and on each sample, 10 measurements were performed in different regions homogeneously distributed on the sample surface. The contact angle was presented as mean ± Standard Deviation (S.D.) from 30 measurements. Statistical analyses were performed using SigmaStat (Jandel Corp., Las Vegas, NV, USA). Multiple comparison procedures were performed by the One Way Analysis of Variance (ANOVA), Student–Newman–Keuls method. *p* values equal to or less than 0.05 were considered significant.

#### 3.2.2. Chemical Composition of the Polymer Surface

The presence of grafted biomolecules, *i.e.*, fibronectin (Fn) and bovine serum albumin (BSA), on the HDPE and LDPE surface was determined by X-ray photoelectron spectroscopy (XPS, Omicron Nanotechnology ESCAProbeP spectrometer) and by immunofluorescence staining. XPS determined the concentration (at.%) of the main elements present in the grafted polymer surface (oxygen, nitrogen, sulfur and carbon).

For immunofluorescence staining, specific primary antibodies against Fn (monoclonal anti-human fibronectin, Sigma-Aldrich, Cat. No. F0791) and BSA (monoclonal anti-bovine serum albumin, Sigma–Aldrich, Cat. No. B2901, St. Louis, MI, USA) were used. The primary antibodies were diluted in PBS to a concentration of 1:200 and were applied to HDPE and LDPE samples modified in plasma and subsequently grafted with Fn or BSA, and were also applied to the non-modified control samples. The samples were incubated with primary antibodies overnight at 4 °C. After rinsing with PBS, the secondary antibody, *i.e.*, goat anti-mouse F(ab’)2 fragments of IgG (H + L), conjugated with Alexa Fluor^®^ 488 (Molecular Probes, Invitrogen, Cat. No. A11017, Eugene, OR, USA), was diluted in PBS to a ratio of 1:400, and was added to the samples for 1 hour at RT. As a staining control, the secondary antibody was applied to samples which had not been treated with the primary antibodies before. The samples were then rinsed twice in PBS. The fluorescence intensity was evaluated from 10 randomly chosen fields homogeneously distributed on the material surface using an epifluorescence microscope (IX 51, Olympus, Tokyo, Japan, obj. 10×) equipped with a digital camera (DP 70, Olympus, Tokyo, Japan) at the same exposure time for all experimental groups (1.2 s). The data was presented as mean ± Standard Error of Mean (S.E.M.). Statistical analyses were performed using SigmaStat (Jandel Corp., Las Vegas, NV, USA). Multiple comparison procedures were made by the One Way Analysis of Variance (ANOVA), Student–Newman–Keuls method. *p* values equal to or less than 0.05 were considered significant.

#### 3.2.3. Surface Morphology and Roughness

The changes in surface morphology and roughness were determined by Atomic Force Microscopy (AFM), using a CP II device (VEECO, Santa Barbara, CA, USA) working in tapping mode. We used an RTESPA-CP Si probe, with spring constant 20–80 N/m. By repeated measurements of the same region (1 × 1 µm), it was proven that the surface morphology did not change after three consecutive scans. The mean roughness value (R_a_) represents the arithmetic average of the deviations from the center plane of the sample.

### 3.3. Cells and Culture Conditions

The samples were sterilized with 70% ethanol for 1 hour, placed into 12-well polystyrene multidishes (TPP, Switzerland, well diameter 2.2 cm) and then air-dried for 12 hours in a sterile environment. The materials were fixed to the bottom of the culture wells by plastic rings in order to prevent them floating in the cell culture media, and were seeded with vascular smooth muscle cells (VSMC), derived from the rat aorta by an explantation method [40]. The cells were used in passage 3. Each well contained 50,000 cells (*i.e.*, about 14,000 cells·cm^−2^) and 3 mL of serum-supplemented or serum-free medium. The serum-supplemented medium was Dulbecco’s modified Eagle’s Medium (DMEM; Sigma, Ronkonkoma, NY, USA, Cat. N° D5648) with 10% of fetal bovine serum (FBS; Sebak GmbH, Aidenbach, Germany) and 40 µg/mL of gentamicin (LEK, Ljubljana, Slovenia), and the serum-free medium was SmGM^®^-2 Smooth Muscle Growth Medium-2 (SmBM; Lonza, Walkersville, MD, USA, Cat. N° CC-3182), supplemented with epithelial growth factor (EGF), fibroblast growth factor-B (FGF-B) and insulin according to the manufacturer’s protocol. The serum-free medium was used in order to enhance the effects on cell adhesion and growth of the chemical functional groups and the biomolecules grafted onto the material surface. This influence can be masked, at least partly, by secondary adsorption of various biomolecules on the polymer surface from the serum supplement of the culture medium. The cells were then cultured for 2, 4 and 6 days in a cell incubator at 37 °C and in a humidified atmosphere with 5% of CO_2_ in the air.

### 3.4. Evaluation of the Cell Number and Cell Spreading Area

On days 2 and 4 after seeding, the cells were rinsed with PBS, fixed with cold 70% ethanol (−20 °C, 5 min), and then stained for 2 hours at RT with a combination of the following fluorescent dyes diluted in PBS: Hoechst #33342 nuclear dye (Sigma-Aldrich, 5 µg·mL^−1^) and Texas Red C_2_-maleimide membrane dye (Molecular Probes, Invitrogen, Carlsbad, CA, USA, Cat. No. T6008, 20 ng·mL^−1^). The cells were counted on microphotographs taken in 10 randomly chosen fields homogeneously distributed on the material surface, using an epifluorescence microscope (Olympus IX 51, Japan, obj. 20×) equipped with a digital camera (DP 70, Japan). This approach was not applicable on day 6, when the cells on some samples reached confluence and started to overlap each another. The cells were therefore detached from the materials by a trypsin-EDTA solution (Sigma-Aldrich, Cat. N° T4174) and were counted using a Vi-CELL XR Analyser (Beckman Coulter, Brea, CA, USA). The pictures taken on day 2 after seeding were also used for measuring the size of the cell spreading area using Atlas software (Tescan Ltd., Brno, Czech Republic).

Two independent samples were used for each time interval and experimental group. Non-modified polyethylene (HDPE or LDPE) and standard cell culture polystyrene wells were used as reference materials.

The quantitative data was presented as mean ± S.E.M. Statistical analyses were performed using SigmaStat (Jandel Corp., USA). Multiple comparison procedures were made by the One Way Analysis of Variance (ANOVA), Student–Newman–Keuls method. *p* values equal to or less than 0.05 were considered significant. The cell numbers obtained on day 2, 4 and 6 after seeding were expressed as the number of cells per cm^2^ (*i.e.*, the cell population density), and were used for constructing growth curves.

## 4. Conclusions

Modification of polyethylene (HDPE and LDPE) with Ar^+^ plasma and grafting with fibronectin and albumin influenced the cell colonization of both polymers. In the serum-supplemented medium, all modifications improved the adhesion and growth of vascular smooth muscle cells in comparison with the unmodified polymer. The cells reached higher final population densities, on an average, on LDPE than on HDPE. In the serum-free medium, grafting with albumin did not support cell colonization due to its non-adhesive properties, which were not masked by the adsorption of cell adhesion-mediating molecules, normally present in the serum. The beneficial effect of the plasma treatment on cell adhesion and growth could be attributed to the formation of new-oxidized structures on the polymer surface, an increase in surface wettability, changes in surface morphology, and enhanced nanoscale roughness. Changes in the physical and chemical properties of the material surface were more apparent on HDPE than on LDPE. The intensity of the fluorescence indicated that HDPE also contained higher quantities of grafted fibronectin and albumin.
